# Determinants of Physical Health of Older People in Iran

**DOI:** 10.1155/2014/507568

**Published:** 2014-10-29

**Authors:** Azar Cheshmberah, Mostafa Hoseini, Davood Shojaee Zadeh, Bijan Moghimi-Dehkordi

**Affiliations:** ^1^Department of Disease Control and Prevention, Iran University of Medical Sciences, Tehran 1985711151, Iran; ^2^Department of Biostatistics, School of Public Health, Tehran University of Medical Sciences, Tehran 1985711151, Iran; ^3^Department of Education and Health Promotion, School of Public Health, Tehran University of Medical Sciences, Tehran 1985711151, Iran; ^4^Department of Health System Research, Shahid Beheshti University of Medical Sciences, Tehran 1985711151, Iran

## Abstract

*Background*. Many of the older people are encountered with physical and mental health problems, chronic diseases, and also living conditions. We aimed to evaluate the disability scores and its associated factors among a sample of older people in Iran. *Material and Methods*. 330 people aged 60–70 years were interviewed about daily living activities and physical functioning using two standard questionnaires. *Results*. According to univariate analysis, aging 66–70 years, being employed, and receiving financial aids were associated with better ADL mean score (*P* < 0.05). Also, being between 66 and 70 years of age, males, being illiterate, being employed, and receiving financial aids were statistically related to higher SF-36 mean scores (*P* < 0.05). Multivariate analyses have shown that higher age and receiving financial aids were related to less need for help and being unemployed with salary was related to higher need for help. However, being illiterate, being employed, and receiving financial aids were significant related factors for better physical functioning. *Conclusions*. Physical health in old people is decreased not only by aging of people but also by other factors such as financial problems and also employment status could decrease physical health of old people regardless of aging.

## 1. Introduction

The aging phenomenon and the growing proportion of the elderly have started in many of developing countries like Iran. While the proportion with 60 years and over comprised 5.4% of total population of Iran in 1975, it is predicted that it will increase to 10.5% in 2025 and to 26% in 2050 [1, 2]. The growing number of older people and their consequent health problems cause many implications on health systems [3]. Many of the older people are encountered with physical and mental health problems, chronic diseases, and also living conditions. In recent years health-related quality of life studies have been of increasing interest by the researchers particularly in developed countries [4–6]. But it has been ignored in many of developing countries. Economical, sociocultural, educational, and health care circumstance may have an effect on quality of life of older people [7]. Approximately 20% of all elderly people are unable to do their daily activities and need help by another person [8]. The economic burden of disabilities of the elderly people is relatively high [9]. A report from Iran has shown that one of four elderly people had extreme to severe disabilities in their lives [10]. Old-age disability was significantly related to sex, age, living status, needing help, marital status, urban/rural residence, drug addiction, duration of addiction, employment status, having regular physical activity, level of education, level of income, health perception, history of hospitalization in the last year, and having multiple diseases [11, 12]. It seems that a lot of aspects of this issue are unknown in our country. The present study was conducted to evaluate the physical health and its associated factors in a random sample of older people in Kermanshah city, west of Iran. It is hoped that the findings of this study are useful for the elderly health care providers and policy makers in Iran and similar communities.

## 2. Material and Methods

This study was conducted with cross-sectional design between April and September 2011, in Kermanshah city (west of Iran). According to sample size formula with the following assumptions SD = 25.4 (based on pilot study on 30 cases), *α* = 0.05, and precision = 3, at least a sample of 275 people is needed. Two-stage sampling was used in this study. In the first step, two elderly care centers were randomly selected and then 330 peoples aged 60–70 years referring to these centers were selected with convenience sampling. Elderly care centers in Iran, public and private, are governed by the State Welfare Organization. After explaining the aims of the study and obtaining informed consent, all participants were interviewed via face to face method and in some cases with telephone by trained investigators using standard questionnaires. The protocol of study was approved by the Ethics Committee of Tehran University of Medical Sciences.

For collecting required data, three questionnaires were used. (1) Sociodemographic details included age, gender, marital status, educational level, employment status, having child, living with children, medical insurance, and receiving financial aids. (2) Activities of daily living (ADL) measurement assess ability and inability of doing the daily activities using five items (bathing, dressing, toileting, transferring, and eating) [13]. Each item has two levels of response: “yes” or “no.” The scores of five items were summed for each participant and the total was presented as a score out of 5. Higher score indicates less need of help in ADL. (3) Physical functioning subscale from SF-36 (Short Form Health Survey) instrument that included 10 items comprised questions about people's ability to do various moderate and severe activities including

1. limit vigorous activities such as running, lifting heavy objects, or participating in strenuous sports,
2. limit moderate activities such as moving a table, pushing a vacuum cleaner, bowling, or playing golf,
3. limit lifting or carrying groceries,
4. limit climbing several flights of stairs,
5. limit climbing one flight of stairs,
6. limit bending, kneeling, or stooping,
7. limit walking more than a kilometer,
8. limit walking half a kilometer,
9. limit walking 100 meter,
10. limit bathing and dressing yourself.

Each item has a three-level response including “a lot,” “a little,” and “not at all.” Final score was obtained by summing scores across the answers for all items and the total was presented as a score out of 30 [14]. High score indicates better physical functioning. All used scales in this study were validated previously for Persian language [15–17].

All statistical analyses were done using SPSS v.18 software (SPSS, Chicago, IL, USA). The significance of differences was assessed by *t*-test and ANOVA. For indication of determinant factor of physical health multiple linear regression was performed. The results of multivariate analysis were presented as full model and final model. All the variables whose *P* value was under 0.25 in the full model have to be included in the final model in order to measure the simultaneous effects of independent variables. A *P* value < 0.05 was considered statistically significant and all reported *P* values were two-sided.

## 3. Results

In all, 330 elderly people aged from 60 to 69 years old (mean age ± SD: 64.9 ± 3.1 years, 44.8% male) were interviewed.  Table 1 presents the demographic features of study population. 69.1% of the participants were married. A few of the study sample had university education and more than half of them were unemployed without salary. Most of them have at least one child and approximately one-third lived with their children. Only a few people did not have medical insurance. 17.9% have received financial aids from charities.

The overall mean scores ± SD of ADL scale and SF-36 were 4.7 ± 0.75 and 26.8 ± 3.9, respectively. Only 2 participants (0.6%) have no limitation regarding ADL scores. The mean scores of two scales according to demographic variables were shown in Table 1. According to univariate analysis, for those aged between 66 and 70 years, being employed and receiving financial aids were associated with higher ADL mean score and lower need for help (*P* < 0.05). Also, peoples between 66 and 70 years of age, males, being illiterate, being employed and receiving financial aids was statistically related to higher SF-36 mean scores (*P* < 0.05).

In order to indicate determinant factors of physical health, linear regression analysis was done. Before conducting the multivariable analysis, the collinearity between independent variables was tested. The results of regression analysis were presented as full model and final model. As shown in Table 2, only age, being unemployed with salary, and receiving financial aids were significant determinants for ADL scales in the final model. However, the results of final model for the SF-36 scale indicate that being illiterate, being employed, and receiving financial aids were significant related factors for better physical functioning (Table 3).

## 4. Discussion

This study aimed to determine some determinant factors that affected physical health of older people. The physical health of participants was evaluated by using two instruments including ADL questionnaire and physical functioning scale of SF-36 instrument. As previously mentioned, age, employment status, and receiving financial aids were the most important factors related to ADL in both univariate and multivariate methods. Different studies have used different instruments for assessing the aging disabilities. Wu et al. using a six-item scale have reported that age is the most significant predictor of chronic ADL disability [18]. Also, gender, educational level, economic status, current health status, and a number of chronic disease were found as related factors to ADL in the other study from Iran [19]. Adib-Hajbaghery [10] in his study on a sample of 350 elderly people in Kashan city, Iran, by using the World Health Organization Disability Assessment Schedule II (WHODAS II) reported a significant relationship between the disability and sex, age, living style, needing help, marital status, living location, addiction, occupation, level of physical activity, educational status, and having multiple diseases. Our results are consistent with some of the findings from other studies. The mentioned studies have used only univariate statistical analysis. In the absence of multivariate analysis, the confounding variables may be affecting the final results. Also, using different scales for assessing the ADL in old people could cause such differences.

Also, we found that age, gender, educational level, employment status, and receiving financial aids were factors associated with physical functioning (Sf-36) in univariate analysis but after the controlling of confounding variables in multivariate analysis only being illiterate, being employed, and not receiving any financial aids were independently related to physical functioning. Both chronic and acute conditions such as cardiovascular disease, bone fractures, joint diseases, problems in mobility, and hospitalization could cause physical disability in older people [20, 21]. The perception of being unable to do some activities in elderly people has also more negative effects on their quality of life than their real level of disability [21].

Age and gender could affect the levels of disability [11, 22] and the older people and women had poorer health situation including physical function [22–25]. Parahyba et al. [26] reported that old-age disability was significantly related to age, gender, low education, and low income. Hoi et al. [4] using EQ-5D questionnaire have reported a significant association among age, educational level, being household head, working, and financial poverty of older individuals. It has been proposed that women have a longer duration of life lived with disability than men [10]. These two variables were omitted from the final model in our study for SF-36 physical function scale. Our findings have proposed that age and gender were not independently related to aging health and the observed association seen in other studies may be due to confounding effect of other variables.

While use of diverse scales together in order to evaluate both physical profiles of participants is one of the advantages of the present study, our sample was not a representative sample for Iranian elderly people and therefore it should be kept in mind that the results should be interpreted cautiously.

## 5. Conclusion

According to our findings, while some determinant factors related to disabilities in older people are not modifiable, and also regarding the fact that most of the Iranian older people lose their financial ability through getting older they were not able to obtain their health and medical needs, it seems that financial support of old people could decrease the level of their disability. Our results could be considered by the health policy makers and health managers in program planning for old people. Other studies using representative sample and with larger sample size are needed for better understanding of the issue.

## Figures and Tables

**Table 1:** Demographic features of study population.

Variables	Number (%)	Physical health (mean ± SD)			
		ADL	*P* value	SF-36	*P* value
Age					
60–65	152 (46.1)	4.6 ± 0.9	0.005	26.0 ± 4.6	0.002
66–70	178 (53.9)	4.8 ± 0.5		27.4 ± 3.1	
Gender					
Male	148 (44.8)	4.6 ± 0.9	0.129	27.4 ± 3.3	0.009
Female	182 (55.2)	4.8 ± 0.6		26.3 ± 4.3	
Marital status					
Married	228 (69.1)	4.7 ± 0.8	0.968	27.0 ± 3.6	0.078
Other	102 (30.9)	4.7 ± 0.7		26.2 ± 4.4	
Education status					
Illiterate	138 (41.8)	4.8 ± 0.7	0.417	27.7 ± 2.9	0.001
Diploma or lower	171 (51.8)	4.7 ± 0.6		26.0 ± 4.5	
University	21 (6.4)	4.7 ± 0.7		26.2 ± 3.0	
Employment status					
Employed	57 (17.3)	4.9 ± 0.3	0.001	28.6 ± 2.1	<0.0001
Unemployed with salary	102 (30.9)	4.5 ± 1.1		26.1 ± 4.3	
Unemployed without salary	171 (51.8)	4.8 ± 0.6		26.6 ± 4.0	
Having child					
Yes	320 (97)	4.7 ± 0.8	0.726	26.8 ± 3.9	0.425
No	10 (3)	4.8 ± 0.4		25.8 ± 2.7	
Living with children					
Yes	227 (68.8)	4.7 ± 0.7	0.878	26.9 ± 3.9	0.267
No	103 (31.2)	4.7 ± 0.8		26.4 ± 3.9	
Medical insurance					
Have	302 (91.5)	4.7 ± 0.8	0.977	26.8 ± 3.9	0.700
Have not	28 (8.5)	4.7 ± 0.7		26.5 ± 4.4	
Receive financial aids					
Yes	59 (17.9)	4.8 ± 0.7	0.010	27.1 ± 3.7	0.001
No	271 (82.1)	4.5 ± 0.9		25.3 ± 4.3	

**Table 2:** Determinants of ADL in older people under study.

Variables	Full model			Final model (*R* ^2^ = 0.11)		
	*β*	SE(*β*)	*P* value	*β*	SE(*β*)	*P* value
Age	0.234	0.082	0.005	0.209	0.080	0.009
Gender (ref: male)	0.126	0.083	0.129	—	—	—
Marital status (ref: married)	−0.004	0.089	0.968	—	—	—
Education						
Illiterate	0.111	0.084	0.186	—	—	—
Diploma or lower	−0.095	0.083	0.252	—	—	—
University	−0.055	0.169	0.745	—	—	—
Employment status						
Employed	0.256	0.108	0.019	—	—	—
Unemployed with salary	−0.302	0.088	0.001	−0.313	0.086	0.000
Unemployed without salary	0.112	0.082	0.177	—	—	—
Having child	−0.084	0.241	0.726	—	—	—
Living with children	0.014	0.089	0.878	—	—	—
Medical insurance	−0.004	0.148	0.977	—	—	—
Receive financial aids	0.276	0.107	0.010	0.282	0.104	0.007

**Table 3:** Determinants of SF-36 in older people under study.

Variables	Full model			Final model (*R* ^2^ = 0.17)		
	*β*	SE(*β*)	*P* value	*β*	SE(*β*)	*P* value
Age	1.347	0.426	0.002	—	—	—
Gender (ref: male)	−1.123	0.429	0.009	—	—	—
Marital status (ref: married)	−0.004	0.050	0.968	—	—	—
Education						
Illiterate	1.673	0.427	0.000	1.738	0.413	0.000
Diploma or lower	−1.495	0.423	0.000	—	—	—
University	−0.571	0.882	0.518	—	—	—
Employment status						
Employed	2.205	0.557	0.000	1.946	0.544	0.000
Unemployed with salary	−1.019	0.463	0.028	—	—	—
Unemployed without salary	−0.390	0.431	0.366	—	—	—
Having child	1.003	1.256	0.425	—	—	—
Living with children	0.516	0.464	0.267	—	—	—
Medical insurance	−0.298	0.773	0.700	—	—	—
Receive financial aids	1.828	0.553	0.001	1.659	0.537	0.002
